# From Remote Monitoring to Actionable Care: Real-World Implementation of an Interoperable Digital Ecosystem for Peritoneal Dialysis

**DOI:** 10.3390/healthcare14193156

**Published:** 2026-09-23

**Authors:** Angela Rivera, Jasmin Vesga, Mauricio Sanabria, Yolima Alba, Andrea Aldana, Kindar Astudillo, Veronica Saavedra, Glorimar Marrero, Bengt Lindholm, Peter Rutherford

**Affiliations:** 1 Vantive, Deerfield, IL 60015, USA; angela.rivera@vantive.com; 2 Renal Care Services Colombia, Bucaramanga 680011, Colombia; jasmin.vesga@vantive.com; 3 Renal Care Services Latin America, Bogotá D.C. 110221, Colombia; 4 Renal Care Services Colombia, Bogotá D.C. 110221, Colombia; lady.alba@vantive.com (Y.A.); andalmir@gmail.com (A.A.); kindar.astudillo@vantive.com (K.A.); glorimar.marrero@vantive.com (G.M.); 5 Vantive Colombia, Bogotá D.C. 112041, Colombia; veronica.saavedra@vantive.com; 6 Division of Renal Medicine, Karolinska Institutet, 171 77 Stockholm, Sweden; bengt.lindholm@ki.se; 7 Vantive, 8152 Zurich, Switzerland; peter.rutherford@vantive.com

**Keywords:** peritoneal dialysis, remote patient monitoring, digital health, electronic medical records, Sharesource, digital ecosystems

## Abstract

**Highlights:**

**What are the main findings?**

- The integration of the Sharesource remote patient monitoring platform with the Versia electronic medical record system was successfully implemented across three dialysis centers, achieving complete data concordance and sustained operational stability.
- Implementation of the integrated digital ecosystem was related with improved workflow efficiency and supported proactive population health management and timely clinical interventions through automated dashboards and real-time access to patient-generated health data.

**What are the implications of the main findings?**

- Interoperability between remote patient monitoring platforms and electronic medical records may transform fragmented data into actionable clinical insights for routine peritoneal dialysis care.
- Integrated digital solutions may support a shift from reactive care models toward proactive population health management, improving operational efficiency and data-driven clinical decision-making.

**Abstract:**

**Background/Objectives:** Remote patient monitoring (RPM) has expanded the capacity of peritoneal dialysis (PD) programs to supervise home-based therapy; however, its full clinical value may be limited when patient-generated data remain disconnected from electronic medical records (EMRs). This study evaluated the implementation and impact of integrating the Sharesource RPM platform with the Versia EMR system in routine PD care. **Methods:** This multicenter real-world implementation study was conducted between November 2024 and February 2025 across three dialysis centers in Colombia. The integration enabled automated transfer of home treatment-related information from Sharesource to Versia and supported population health management through interactive dashboards. Workflow efficiency was assessed using a before-and-after time and motion analysis, and user experience was evaluated through structured surveys administered 12 weeks after implementation. Analyses were descriptive. **Results:** The integration was successfully implemented across the participating centers, achieving complete data concordance and operational stability. During follow-up of 287 patients, 501 patient-clinic communications, 159 preemptive evaluations, and 122 prescription modifications were recorded, reflecting proactive identification and management of clinical issues. Nursing consultation time decreased from 34 to 26 min per patient, and nephrologist time from 31 to 25 min per patient. Surveys showed high acceptance, supporting proactive clinical decision-making, and greater workflow efficiency. **Conclusions:** Real-world implementation of an interoperable RPM-EMR ecosystem in PD care was feasible, operationally stable, and was related to improvements in workflow efficiency while supporting proactive population health management. By transforming fragmented patient-generated data into actionable clinical insights, this digital ecosystem may support more efficient, data-driven, and proactive models of chronic kidney disease care.

## 1. Introduction

The global burden of kidney failure continues to rise, placing substantial pressure on healthcare systems to deliver high-quality, sustainable, and patient-centered kidney replacement therapies (KRT) [1,2]. In this context, peritoneal dialysis (PD) represents an important home-based treatment modality that supports patient autonomy, treatment flexibility, and continuity of chronic kidney disease care [3,4,5,6]. PD is particularly relevant in Colombia, where 24% of patients receiving dialysis are treated with this modality, highlighting its important role within the national kidney replacement therapy landscape [7]. This substantial use of PD also underscores the relevance of developing scalable digital strategies to support the delivery and monitoring of home-based dialysis care. However, the success of home-based dialysis depends on effective monitoring, timely clinical intervention, and strong coordination between patients and multidisciplinary care teams [4,6].

Recent advances in digital health have transformed the management of PD programs. Remote patient monitoring (RPM) has expanded the capacity of PD programs to supervise therapy performed in the home setting [8,9,10,11,12,13,14]. In automated peritoneal dialysis (APD), RPM platforms enable access to treatment-related information such as adherence, ultrafiltration, blood pressure, body weight, cycler alerts, prescription execution, and session-level performance [8,9,11]. Sharesource™ (Baxter Healthcare, Deerfield, IL, USA) is one of the most widely adopted RPM platforms for APD that enables secure transmission of home cycler data to clinical teams and may support earlier identification of clinical or technical issues [8,9,10,11,12,13,14].

Although RPM has improved access to patient-generated health data, its clinical value may be limited when remote monitoring systems remain disconnected from electronic medical records (EMRs) [15,16,17,18]. In fragmented digital environments, clinicians may need to access multiple platforms, manually reconcile information, and duplicate documentation [15,19]. As a result, large volumes of patient-generated data may contribute to information overload rather than actionable clinical insight, adding to the time devoted to documentation which constitutes a major portion of the workday time for physicians and nurses [20]. This fragmentation reduces workflow efficiency, increases the administrative burden, and limits the ability of clinical teams to use RPM data proactively in routine care [17,19].

Healthcare interoperability between RPM platforms and nephrology-specific EMRs may represent the next stage of digital transformation in chronic kidney disease care [16,19,21,22]. By embedding remote monitoring data directly into the patient record and pairing these data with interactive dashboards, integrated digital ecosystems can support patient prioritization, risk surveillance, and population health management [16,22,23]. However, real-world evidence remains limited regarding the implementation, workflow impact, operational performance, and user experience of RPM-EMR integration in routine PD care [16,19].

To address this gap, the Renal Care Services network implemented an interoperable digital ecosystem integrating the Sharesource RPM platform with the Versia EMR across three dialysis centers in Colombia. Unlike previous approaches in which RPM data are reviewed through standalone platforms or dashboards, this integration embeds PD treatment data within the routine EMR workflow, enabling clinicians to review remote treatment information alongside longitudinal clinical data within a unified digital environment. The integration combined automated data transfer with interactive dashboards designed to support clinical decision-making and proactive population health management for home-based APD care. This study aimed to evaluate the real-world implementation and operational impact of the Sharesource-Versia integration in routine APD care, with emphasis on technical performance, workflow efficiency, population health management, and user experience.

## 2. Materials and Methods

### 2.1. Study Design and Setting

This multicenter, real-world implementation study used a before-and-after design to evaluate the deployment among patients receiving treatment with automated peritoneal dialysis in their homes of an interoperable digital ecosystem integrating the Sharesource remote patient monitoring platform with the Versia electronic medical record system. The study was conducted between November 2024 and February 2025, following a 12-month software development and implementation process, across three dialysis centers belonging to the Renal Care Services network in Colombia. All participating centers provided routine care for patients receiving automated peritoneal dialysis and used Sharesource as part of their remote monitoring workflow. The centers operated with a nurse-to-patient ratio of approximately 1:45 and a nephrologist-to-patient ratio of approximately 1:140. All adult patients receiving automated peritoneal dialysis and being monitored through Sharesource during the implementation period were eligible for inclusion. No additional inclusion or exclusion criteria were applied, as the objective was to assess implementation under routine clinical practice conditions. The integrated population management dashboards included 287 patients across the participating centers. Post-implementation time-and-motion observations and usability assessments were conducted 12 weeks after integration, allowing clinicians sufficient time to become familiar with the system and incorporate it into routine clinical practice before formal evaluation.

### 2.2. Development of the Interoperable Digital Ecosystem

The intervention consisted of the development and implementation of an interoperable digital ecosystem connecting Sharesource and Versia. The objective was to automate the transfer of patient-generated treatment data from the remote monitoring platform into the electronic medical record and to make these data available for routine clinical review, documentation, and population health management. A dedicated peritoneal dialysis module, referred to as the PD Treatment module, was developed within Versia. This module enabled clinicians to access remote monitoring information directly within the patient record, including dialysis prescriptions, treatment adherence, ultrafiltration parameters, blood pressure measurements, body weight records, cycler alerts, treatment execution data, and session-level performance indicators.

The implementation followed five sequential phases: needs assessment, software development and integration architecture, validation and regulatory review, user training, and operational deployment (Figure 1). The needs assessment focused on existing clinical workflows, information gaps, manual documentation requirements, and barriers related to the use of disconnected systems. Software development included definition of the integration architecture, data model, data transfer processes, and user-facing clinical views within the EMR.

Data exchange between Sharesource and Versia was implemented through proprietary REST APIs connected by an intermediate integration layer. This layer retrieved data from the Sharesource API, transformed and mapped the information to the format required by Versia, and transferred the resulting data through the Versia REST API. Data synchronization was performed asynchronously through scheduled background processing using internal queues, with OAuth2-based authentication for both systems.

Before deployment, the integration underwent software quality assurance, cybersecurity assessment, user acceptance testing, and formal validation procedures. Validation activities were designed to confirm data integrity, traceability, auditability, system reliability, and concordance between information displayed in Sharesource and data transferred into Versia. These activities included review and approval of user and system requirements, architecture and design documentation, cybersecurity and risk assessments, regulatory and quality reviews, traceability verification, functional testing, integration testing, regression testing, and defect management. The validation approach followed principles derived from the U.S. Food and Drug Administration 21 CFR Part 11 framework for electronic records and electronic signatures [24]. Access controls, audit trails, and electronic signature functionality were included in the validation framework to ensure authorized system access, traceability of system activities, and integrity of electronic records. Data concordance was assessed using predefined test cases designed to confirm that information was transferred accurately, completely, and reliably between Sharesource and Versia. Acceptable concordance was defined as the successful completion of all planned validation activities and test cases, demonstrating that transferred information remained complete, accurate, and consistent with expected results. Any identified discrepancies were investigated and resolved before implementation. Operational readiness was confirmed before routine clinical use. Following validation, users received training on the PD Treatment module, dashboard interpretation, and standardized workflows for reviewing integrated data [16,22].

### 2.3. Population Health Management Framework

After implementation, data transferred from Sharesource were combined with clinical information available in Versia and visualized through interactive dashboards developed using Microsoft Power BI v2.157.1354.0. These dashboards supported both individual patient review and population-level surveillance across the automated peritoneal dialysis program.

The dashboards monitored key clinical and operational indicators, including treatment adherence, ultrafiltration performance, blood pressure control, body weight trends, dialysis prescription changes, cycler alerts, patient-clinic communications, preventive interventions, and indicators potentially associated with treatment discontinuation risk. Patients requiring clinical attention were identified using predefined monitoring criteria embedded within the dashboards, including missed treatments, ultrafiltration or dry-weight abnormalities, blood pressure values outside target ranges, catheter-related issues, cycler malfunctions, suspected infectious complications, poor metabolic control, and signals suggesting increased risk of therapy discontinuation.

The population health management framework was designed to support, not replace, clinical judgment. Dashboard-generated insights were used by clinical teams to prioritize patients for outreach, preventive clinical assessment, prescription review, and individualized treatment optimization.

### 2.4. Time-And-Motion Analysis

Workflow efficiency was evaluated using a structured time-and-motion analysis conducted before and after implementation of the integrated platform. During the pre-implementation phase, conducted from 5 November to 15 November 2024, fifteen healthcare professionals, including nephrologists (n = 6) and peritoneal dialysis nurses (n = 9), were observed during 245 patient-care activities over 133 h. During the post-implementation phase, conducted from 3 February to 17 February 2025, the same methodology was applied to 208 patient-care activities observed over 114 h. The observation periods were selected to coincide with scheduled nephrology consultation days for the relevant PD population at the participating clinics; other working days were allocated to clinical activities involving other patient populations, including hemodialysis and nephroprotection. All observations in both periods were conducted by the same external workflow assessment consultant using a continuous direct-observation methodology. Because a single observer performed all assessments, inter-rater reliability testing was not applicable. The observer was aware of the study objectives and implementation phase, as blinding was not feasible in the context of this real-world implementation assessment. Activities were categorized according to a predefined classification framework established before the observational assessment and applied consistently during both study periods. Value-added activities were defined as tasks directly contributing to patient assessment, clinical decision-making, treatment planning, patient education, or care coordination. Non-value-added activities were defined as workflow tasks that did not directly contribute to these functions and represented potentially reducible process burden, including duplicate documentation, manual data reconciliation, searching for clinical information, and navigation across disconnected digital systems. Clinicians were aware that workflow observations and timing were being conducted because direct observation was required for continuous activity timing. Because multiple activities were observed within the workflows of the same healthcare professionals, activity-level observations were not assumed to be statistically independent for inferential testing. The time-and-motion analysis was therefore used primarily as a descriptive workflow assessment. Workflow outcomes included total consultation time per patient for nurses and nephrologists, time dedicated to reviewing Sharesource information, report-generation time, and the proportion of time spent on value-added activities.

### 2.5. User Experience Assessment

User experience was assessed using structured surveys administered to nephrologists and peritoneal dialysis nurses 12 weeks after implementation of the integrated platform. Responses were anonymized before analysis. The survey evaluated domains related to overall experience, usability and design, system performance and interoperability, clinical functionality, workflow support, documentation and reporting, and perceived clinical value. Responses were collected using a 7-point Likert scale, where higher scores indicated more favorable perceptions (Supplementary Materials). Survey responses were summarized separately for nephrologists and nurses using means and standard deviations. Domain-level findings were used to identify perceived strengths, limitations, and opportunities for further platform optimization.

### 2.6. Statistical Analysis

Analyses were primarily descriptive. Continuous variables are presented as means, and categorical variables as frequencies and percentages. Changes observed between the pre-implementation and post-implementation periods are reported as absolute and relative differences. Because this was an implementation-focused study, analyses were intended to describe operational and clinical impact rather than formally test hypotheses. For time-and-motion outcomes, before and after implementation measurements were compared at the healthcare-professional level using paired statistical tests. The distribution of within-participant differences was evaluated with Wilcoxon signed-rank tests to continuous outcomes and McNemar’s tests to categorical outcomes. A two-sided *p*-value < 0.05 was considered statistically significant. Statistical analyses were performed using Stata 16^®^ (StataCorp LLC, College Station, TX, USA).

## 3. Results

The primary implementation outcomes included technical integration success, data concordance, workflow efficiency, and user experience. Secondary outcomes included population health management indicators, consultation times, report generation time, and proactive clinical interventions.

### 3.1. Technical Implementation Outcomes

The interoperable RPM-EMR ecosystem was successfully implemented across the three participating centers following a 12-month software development and implementation process. Deployment was completed without interruption of routine clinical care after successful completion of software quality assurance, cybersecurity assessment, software validation, and change management procedures.

Software validation demonstrated complete concordance between records displayed in the Sharesource remote monitoring platform and those transferred to the Versia electronic medical record. No data loss, synchronization failures, or transfer errors were identified during implementation, and the integrated platform maintained continuous operational stability without unplanned downtime throughout the study period.

Successful implementation required addressing several technical and organizational challenges, including credential management, access-control mechanisms, architectural compatibility, software dependencies, version control, and regulatory compliance. These activities ensured system quality, traceability, auditability, and scalability while supporting secure exchange of patient-generated health data between both platforms.

Following implementation, operational performance metrics showed substantial improvements, including a marked reduction in report-generation time and reduced need for clinicians to manually retrieve information from separate digital systems. The PD Treatment module enabled direct access to dialysis prescriptions, data on treatment adherence, ultrafiltration, blood pressure, and body weight, cycler alarms, and treatment performance indicators from within the electronic medical record (Figure 2). Collectively, these findings demonstrate that implementation extended beyond technical interoperability by establishing a robust, scalable, and clinically deployable digital ecosystem capable of supporting routine clinical workflows and population health management in an automated peritoneal dialysis program.

### 3.2. Workflow Efficiency Outcomes

Following implementation of the interoperable RPM–EMR ecosystem, substantial improvements in workflow-efficiency metrics were observed, coinciding with automated aggregation of remote patient monitoring and clinical information within a unified digital environment. Report generation time, defined as the total time required for clinical staff to retrieve, consolidate, review, and prepare patient monitoring information from remote patient monitoring platforms and the electronic medical record for clinical assessment, decreased from 70 min to 15 min, representing an approximately 80% reduction. The integrated workflow reduced the need for manual data consolidation across multiple digital platforms and enabled clinicians to access comprehensive treatment information directly within the Versia electronic medical record through the PD Treatment module.

The interoperable ecosystem streamlined routine clinical workflows by providing immediate access to dialysis prescriptions, treatment adherence, ultrafiltration, blood pressure, body weight, cycler alarms, and treatment performance indicators within a single interface. Interactive dashboards developed using Microsoft Power BI further facilitated rapid identification of patients requiring clinical attention and supported prioritization of daily clinical activities without the need to navigate between independent information systems (Figure 3).

Together, these findings demonstrate more efficient access to integrated patient information during routine clinical care.

### 3.3. Population Health Management Outcomes

The integrated analytics environment enabled proactive identification of patients requiring clinical intervention and supported population-level management across the APD program. During the study period, 501 patient-clinic communications were recorded. The most frequent triggers were missed home dialysis sessions (n = 106; 21.2%), PD catheter-related issues (n = 99; 19.8%), ultrafiltration or dry-weight abnormalities (n = 86; 17.2%), vital signs outside predefined target ranges (n = 76; 15.2%), follow-up issues (n = 44; 8.8%), and cycler malfunctions (n = 43; 8.6%) (Table 1).

A total of 159 preemptive clinical visits were performed in 99 patients following identification of clinically relevant findings through the integrated monitoring systems. The most common reasons for these evaluations included ultrafiltration or dry-weight abnormalities (27.0%), PD catheter-related complications (19.5%), vital signs outside target ranges (17.0%), suspected peritonitis or exit-site infection (11.3%), cycler malfunctions (7.5%), and poor metabolic control (5.7%). In contrast, only 34 emergency department visits were recorded among 28 patients during the study period. The most common causes were heart failure (n = 6), respiratory diseases (n = 6), other cardiovascular events (n = 4), skin disorders (n = 4), cerebrovascular events (n = 3), and gastrointestinal diseases (n = 3).

Additionally, 122 dialysis prescription modifications were implemented in 93 patients. These changes were primarily related to dialysis adequacy, ultrafiltration performance, and PD catheter function, supporting individualized treatment optimization.

### 3.4. Time-And-Motion Outcomes

Implementation of the interoperable RPM–EMR ecosystem was associated with measurable improvements in consultation efficiency for both nurses and nephrologists. Overall consultation time during the study period decreased from 34 to 26 min per patient for nurses, representing a 23.5% reduction, and from 31 to 25 min per patient for nephrologists, representing a 19.4% reduction, following implementation of the integrated platform.

The greatest efficiency gains were observed in activities related to review and interpretation of remote monitoring data. Among nurses, the average time dedicated to reviewing Sharesource information decreased from 4.4 to 2.0 min per patient (54.5% reduction), while among nephrologists this time decreased from 2.5 to 1.0 min per patient (60.0% reduction). Direct integration of RPM data within the electronic medical record reduced the need to navigate multiple digital platforms and facilitated more efficient access to treatment information during routine consultations (Table 2).

Value stream mapping demonstrated a consistent shift toward value-added clinical activities. The proportion of value-added time increased from 89% to 90% during nephrology consultations and from 90% to 94% during nursing consultations, reflecting reductions in duplicate documentation, data reconciliation, and navigation across disconnected information systems.

### 3.5. User Experience Outcomes

Overall, the integrated RPM-EMR ecosystem was well accepted by both nephrologists and nurses, with favorable ratings across domains related to usability, clinical functionality, workflow support, and perceived clinical value. Nephrologists assigned the highest scores to data accuracy, patient triage capabilities, review of key clinical parameters, and patient assessment functions, reflecting the platform’s utility for clinical decision-making during routine practice. Nurses reported the highest ratings for treatment adherence monitoring, proactive identification of patient issues, workflow support, and prescription tracking, highlighting the value of the integrated platform for longitudinal patient follow-up and population surveillance.

Across both professional groups, system configurability and customization received the lowest ratings, identifying specific limitations of the current implementation and opportunities for future platform refinement, while overall acceptance of the integrated ecosystem remained favorable (Table 3).

## 4. Discussion

This multicenter real-world implementation study demonstrates that an interoperable RPM-EMR ecosystem can be successfully integrated into routine peritoneal dialysis care. The implementation achieved complete data concordance, operational stability, and incorporation of patient-generated health data into clinical workflows. Beyond technical integration, the ecosystem supported proactive clinical management through interactive dashboards and population-level analytics, enabling more efficient and data-driven models of chronic kidney disease care [16,19,21,23].

Previous studies have consistently demonstrated that RPM improves treatment adherence, patient engagement, technique survival, and clinical outcomes in patients receiving automated peritoneal dialysis [10,11,12,13,14]. Our findings extend this evidence by addressing an implementation gap: how remote monitoring data can become usable in routine clinical workflows. The results suggest that the value of RPM may be substantially enhanced when patient-generated health data are integrated directly into the EMR, reducing information fragmentation and improving access to clinically relevant information at the point of care [16,23].

Although interoperability is often discussed as a technical objective, its clinical value lies in transforming dispersed patient-generated information into actionable intelligence [16,18,25,26,27]. In this study, automated data transfer reduced the need for manual reconciliation and enabled clinicians to review dialysis prescriptions, treatment adherence, ultrafiltration, blood pressure, weight, cycler alerts, and treatment performance indicators within a unified digital environment.

The integrated dashboards further enabled risk surveillance, patient prioritization, and timely interventions. These findings support the concept that interoperability should be understood not only as data exchange, but as a clinical enabler that embeds patient-generated data into decision-making processes and supports coordinated chronic care delivery [28,29,30,31]. Furthermore, the interoperable RPM-EMR ecosystem reduces the time for finding and documenting observations and medical actions; the time required for documentation represents a major burden for the healthcare work force [20].

A key benefit of the integrated platform was its support for proactive population health management. The dashboard enabled surveillance of 287 patients receiving automated peritoneal dialysis and supported clinic-initiated communication, preemptive clinical visits, and prescription modifications. The predominance of clinic-initiated communication suggests a shift from reactive care to proactive outreach, while preemptive visits and prescription changes indicate that integrated data were actively used to identify emerging problems and optimize treatment [9,31]. However, these findings represent process outcomes and should not be interpreted as evidence of improved clinical outcomes. Whether these proactive interventions translate into reductions in hospitalization, peritonitis, technique failure, or other clinically relevant outcomes requires evaluation in longer-term studies.

The implementation also generated measurable operational benefits. Report generation time decreased markedly, consultation time decreased for both nurses and nephrologists, and time dedicated to reviewing RPM data was reduced by more than half. These findings are relevant because digital health tools may fail to achieve adoption if they increase complexity or add administrative burden. In this study, integration reduced friction in clinical workflows by limiting duplicate documentation, manual data reconciliation, and reducing time demand for navigation across disconnected platforms [31,32]. The time needed for clinical documentation versus direct patient care is a major problem in healthcare and may contribute to low job satisfaction [33]. The ability of the interoperable RPM-EMR ecosystem to improve efficiency and facilitate direct patient care is therefore a major advantage. Given the uncontrolled before-and-after design, the observed differences in workflow metrics should be interpreted as associations with implementation rather than effects attributable exclusively to the digital ecosystem.

User experience findings further support implementation feasibility. Nephrologists valued data accuracy, triage capabilities, review of key clinical parameters, and patient assessment functions, reflecting the platform’s utility for clinical decision-making. Nurses valued adherence monitoring, proactive identification of patient issues, prescription tracking, and longitudinal follow-up, highlighting the importance of integrated data for population surveillance. Differences between professional groups were observed for specific functions, with nurses reporting lower ratings for patient triage and dashboard/patient-summary generation, suggesting profession-specific workflow and usability requirements that warrant further evaluation. Lower ratings for system configurability and customization identify practical opportunities for further refinement and underscore the need for iterative end-user feedback, as limitations in these functions may affect long-term user acceptance and sustained adoption.

Successful implementation requires more than technical connectivity. It depended on workflow mapping, cybersecurity and validation processes, user training, change management, and alignment with clinical priorities. These implementation lessons are important for scalability because interoperable digital ecosystems must be adapted to local workflows, data governance requirements, and clinical decision-making processes. The personnel time and costs associated with development and implementation were not prospectively collected and therefore could not be reliably quantified. Consequently, the feasibility of replication in other settings should be considered in the context of locally available technical expertise, digital infrastructure, and implementation resources.

This study has several strengths, including its multicenter real-world design, evaluation under routine clinical practice conditions, and assessment of multiple outcome domains, including technical performance, workflow efficiency, population health management, and user experience. Limitations should also be acknowledged. The study was conducted within a single healthcare network in Colombia, which may limit its generalizability. The before-and-after design lacked a concurrent control group and therefore does not permit causal attribution of the observed workflow changes exclusively to the intervention. The follow-up was relatively short, and the analysis was descriptive rather than powered for clinical outcomes. In addition, formal economic evaluation, patient-reported outcomes, and long-term outcomes such as hospitalization, peritonitis, technique survival, and cost-effectiveness were not assessed. Finally, because healthcare professionals were aware that direct workflow observations were being conducted and the observer was aware of the study objectives and implementation phase, Hawthorne and observer effects cannot be excluded. The use of the same external observer, continuous timing methodology, and predefined activity-classification framework in both periods provided consistency in data collection but does not eliminate these potential sources of bias. In addition, increasing staff familiarity with the integrated platform over time may have contributed to the observed improvements in workflow efficiency. Awareness of being observed may also have temporarily influenced work practices, potentially leading to an overestimation of the efficiency gains observed in the post-implementation period. Another limitation of the present study is that information on the time, human resources, and financial investment required for the development and implementation of the project was not systematically collected, although such information would have been valuable for assessing the resources required to replicate similar implementations in other healthcare settings.

Accordingly, the observed changes in workflow metrics should be interpreted as temporal associations following implementation rather than definitive causal effects of the integrated platform. Future studies should evaluate longer-term clinical outcomes, economic impact, patient and caregiver experience, and scalability across diverse dialysis programs and healthcare systems. Looking ahead, interoperable RPM-EMR infrastructures may also provide a foundation for emerging artificial intelligence (AI)-enabled kidney care. By integrating longitudinal treatment and clinical data within a unified environment, these ecosystems could support future applications such as AI-assisted patient monitoring, automated clinical summarization, risk prediction, clinical decision support, medication safety monitoring, and more personalized kidney care [34]. Beyond workflow optimization, integrated RPM-EMR ecosystems may also support medication safety monitoring in chronic kidney disease by combining remote monitoring and clinical data within a single environment. This integrated information may facilitate earlier identification of potential medication-related issues and support future predictive analytics and pharmacovigilance strategies. In this context, our findings on improved data access and proactive identification of clinical issues suggest that interoperability may provide a foundation for more personalized and proactive safety monitoring [35]. However, these applications will require rigorous validation, appropriate human oversight, and careful consideration of data quality, transparency, and patient safety before routine clinical implementation.

## 5. Conclusions

In conclusion, RPM-EMR interoperability was related to improved integration of remote patient monitoring data into clinical workflows, including reduced information fragmentation, high data concordance, improved access to patient-generated health data, and perceived workflow efficiencies. The integrated environment appeared to support a more proactive approach to peritoneal dialysis care through patient prioritization, clinic-initiated communication, earlier identification of clinical issues, and individualized prescription adjustments. These real-world findings suggest that interoperable digital ecosystems may support the use of remote monitoring data to inform clinical decision-making. Although causality cannot be inferred from this before-and-after study design, the observed findings following implementation warrant further evaluation in controlled studies.

## Figures and Tables

**Figure 1:**
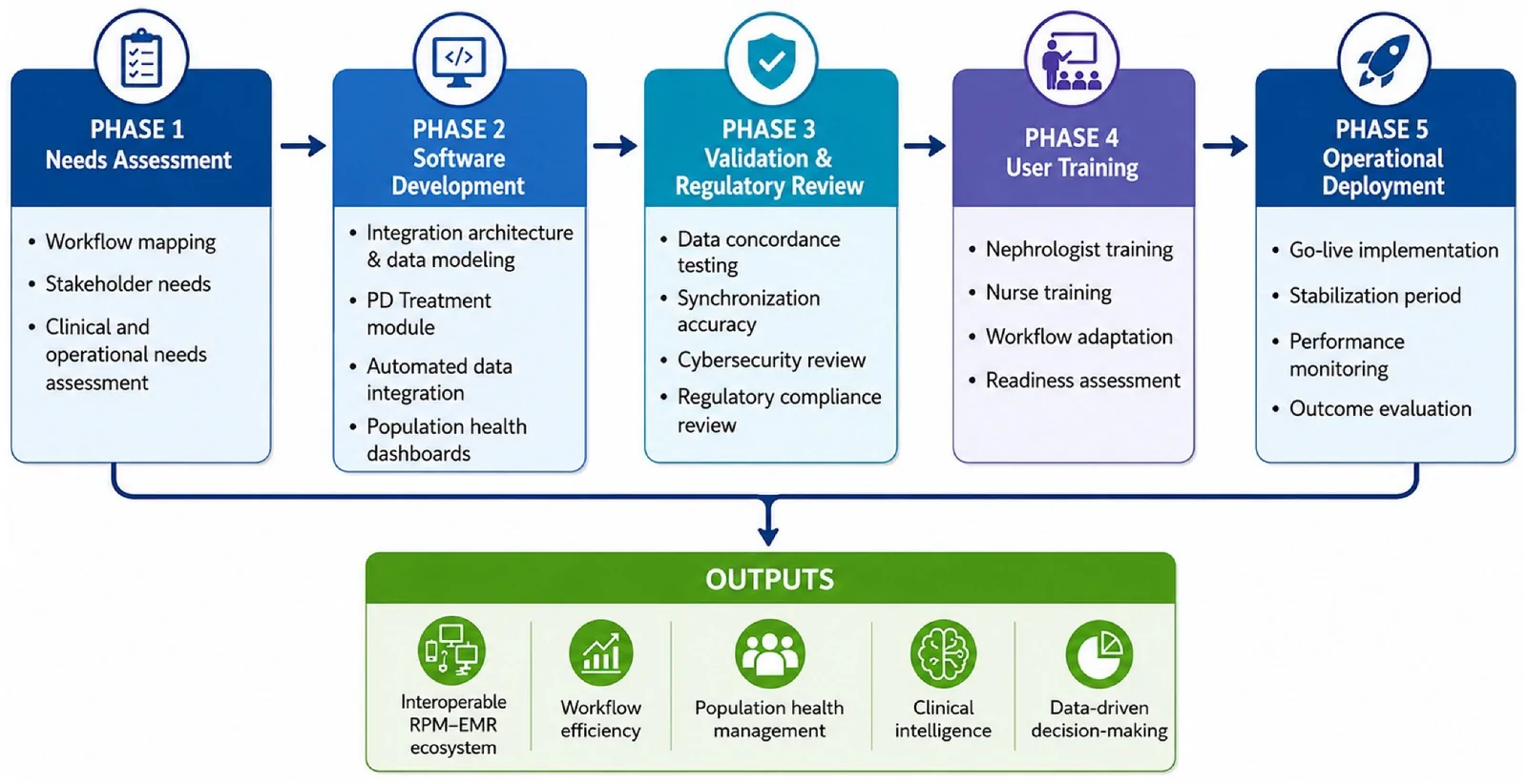
Five-phase implementation framework for the Sharesource–Versia integration. The figure summarizes the sequential implementation framework used to develop, validate, and deploy the interoperable RPM–EMR ecosystem.

**Figure 2:**
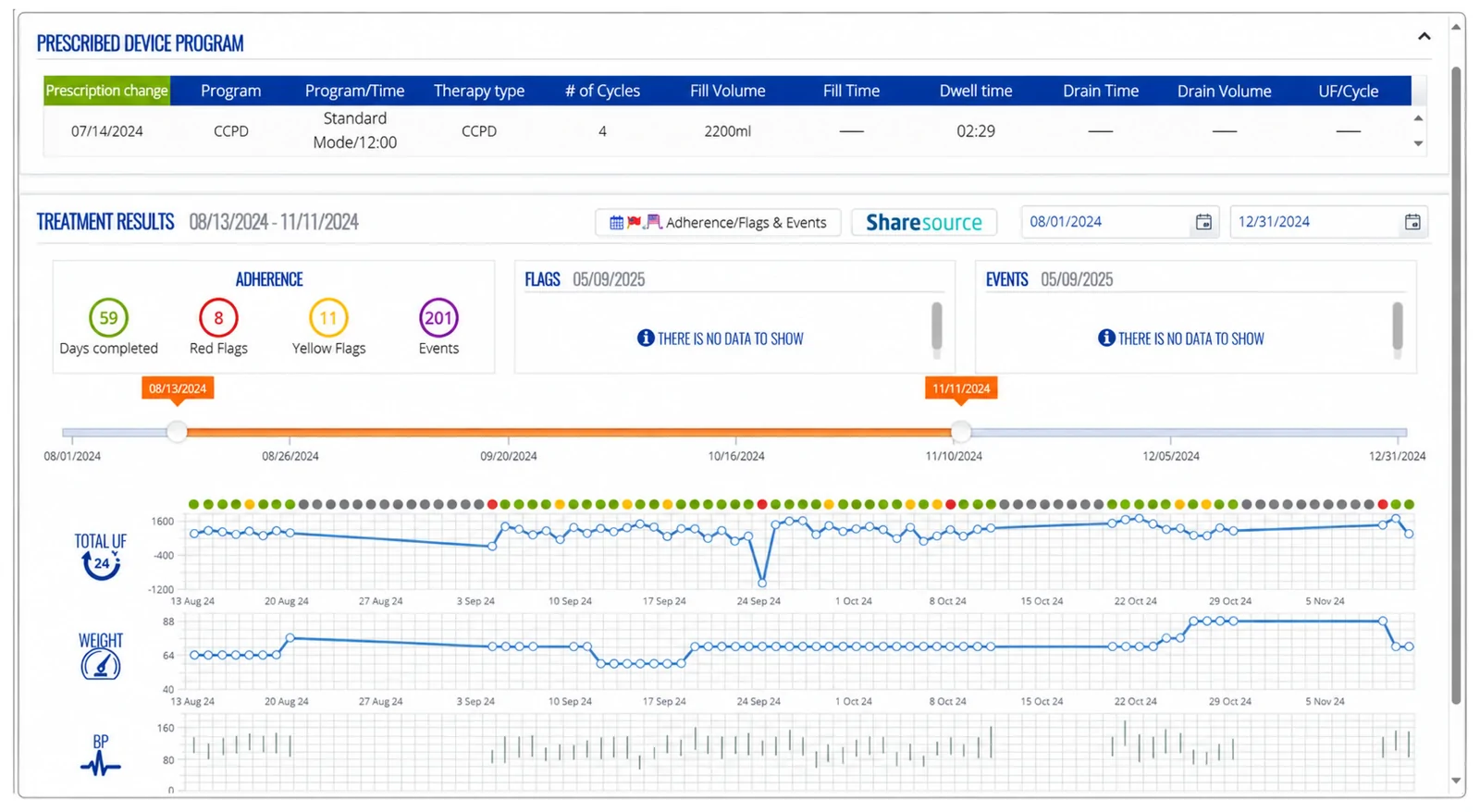
Integration of Sharesource data into Versia. The figure illustrates the integration between Sharesource and Versia, including prescribed device program parameters, treatment adherence, alerts, events, and longitudinal clinical data such as total ultrafiltration, body weight, and blood pressure.

**Figure 3:**
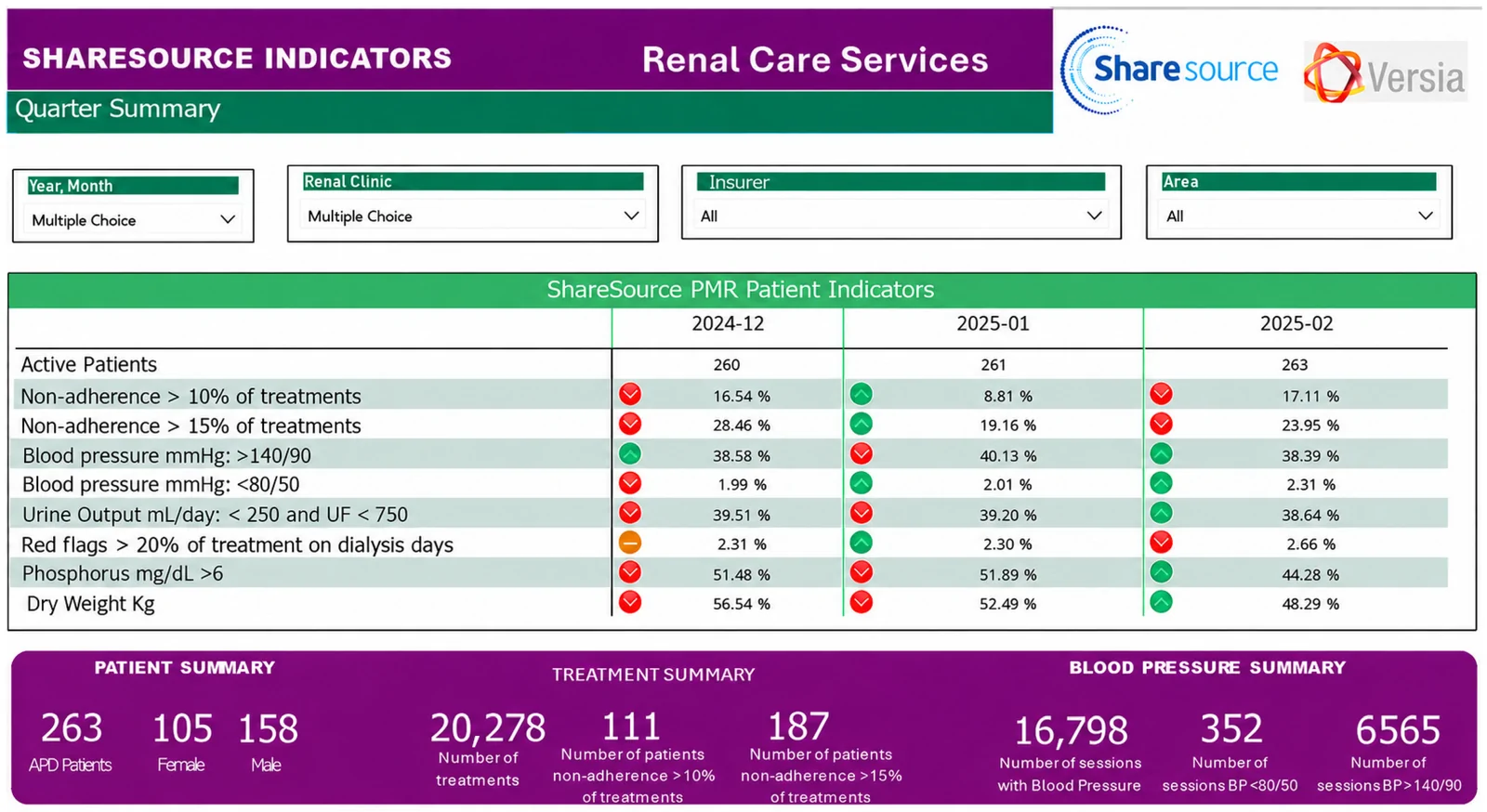
Interactive population health dashboard integrating Sharesource remote monitoring data with Versia electronic medical record data. Indicator performance is classified according to predefined thresholds and displayed using a traffic-light system: green indicates performance within target, orange indicates values requiring closer monitoring, and red indicates performance outside the established target requiring corrective action.

**Table 1:** Population health management outcomes following implementation of the integrated RPM-EMR ecosystem.

Domain	Measure	Result
Population surveillance	Patients monitored	287
Risk identification	Patient-clinic communications	501
	Clinic initiated	463 (92.4%)
	Patient initiated	38 (7.6%)
Clinical prioritization	Preemptive clinical visits	159
Treatment optimization	Prescription modifications	122
Healthcare utilization	Emergency department visits	34

**Table 2:** Time-and-motion analysis before and after Sharesource–Versia integration.

Activity	Before Integration	After Integration	Relative Change	*p*-Value
Nursing consultation time (minutes/patient)	34	26	−23.5%	<0.001
Nephrologist consultation time (minutes/patient)	31	25	−19.4%	<0.001
Nurse RPM review time (minutes/patient)	4.4	2	−54.5%	<0.001
Nephrologist RPM review time (minutes/patient)	2.5	1	−60.0%	0.007
Value-added nursing activities (%)	90	94	+4.4%	0.798
Value-added nephrology activities (%)	89	90	+1.1%	0.954

**Table 3:** Nephrologist and nurse ratings of the Versia-Sharesource PD Treatment module across key domains.

Item	Nephrologists, N = 6 Mean ± SD	Nurses, N = 9 Mean ± SD
**Overall Experience**		
Comfortability using Versia-Sharesource integration	6.2 (0.9)	5.7 (1.0)
Likelihood to recommend the PD module to colleagues	6.0 (1.3)	5.8 (0.7)
**Usability & Design**		
Easy to navigate	6.5 (0.5)	5.8 (1.3)
Configurable by the user	1.8 (1.3)	1.0 (0.0)
Customizable	1.7 (1.8)	1.4 (1.3)
**System Performance & Integration**		
Well integrated/good interoperability	5.5 (1.6)	5.3 (1.3)
Data accuracy	6.7 (0.5)	4.8 (1.9)
**Clinical Functionality**		
Ability to track adherence	6.7 (0.5)	5.9 (1.3)
Ability to track flags and events	5.8 (1.5)	5.7 (1.7)
Ability to track key parameters (UF, weight, blood pressure)	6.7 (0.5)	5.6 (1.6)
Ability to triage patients	6.7 (0.5)	2.7 (1.6)
Ability to review and assess patients	6.6 (0.5)	5.0 (1.0)
Ability to proactively identify patient issues	6.0 (1.2)	5.6 (1.3)
Ability to review PD cycler data	6.2 (1.2)	5.4 (1.5)
**Workflow Support**		
Supports tracking of prescriptions and program changes	5.5 (2.1)	5.4 (1.6)
Simplifies PD workflow	5.5 (1.8)	3.7 (1.1)
Saves time	5.7 (1.8)	4.8 (1.3)
**Documentation & Reporting**		
Printing documents	6.5 (0.8)	5.2 (1.5)
Improves patient review and assessment (clinical & treatment data)	6.0 (1.3)	5.1 (1.2)
Improve documentation in Versia	4.7 (2.1)	5.6 (1.5)
Generates dashboards/patient summaries	5.0 (2.1)	2.4 (1.7)
**Perceived Clinical Value**		
Improves tracking of prescriptions and program changes	5.0 (2.4)	5.8 (1.6)
Improves tracking of adherence	6.0 (1.5)	5.3 (1.1)
Improves tracking of key parameters (UF, weight, blood pressure)	6.7 (0.5)	5.2 (1.6)
Supports discussion with patients about treatment status and progress	6.2 (1.2)	5.6 (1.3)

Likert-scale responses were scored from 1 to 7, with higher scores reflecting more positive evaluations of usability, usefulness, comfort with use, and likelihood of recommending the Versia PD Treatment module.

## Data Availability

The data presented in this study are available on request from the corresponding author. The data are not publicly available due to privacy and ethical restrictions.

## Supplementary Materials

The following supporting information can be downloaded at: https://www.mdpi.com/article/10.3390/healthcare14193156/s1, Assessment of clinical workflow and user experience following the integrating of Sharesource-versia.
