# Supplementary material for: From Remote Monitoring to Actionable Care: Real-World Implementation of an Interoperable Digital Ecosystem for Peritoneal Dialysis
Source: Healthcare (Basel). 2026 Sep 23;14(19):3156. doi: 10.3390/healthcare14193156 (PMC13665226; doi:10.3390/healthcare14193156)
Supplement: Supplementary file 1 [file healthcare-14-03156-s001.zip › healthcare-4497936-supplementary.pdf]

Supplementay Material

Assessment of Clinical Workflow and User Experience Following the Integration of Sharesource-Versia

We would now like to evaluate how the integration of Sharesource-Versia has impacted the peritoneal dialysis (PD) workflow and the management of PD patients, compared with the previous workflow when this integration was not available.

Please answer the following questions based on your experience and professional perception. There are no right or wrong answers; we are interested in understanding your perspective regarding the impact of the integration on your daily clinical practice.

Please rate the following features of the Sharesource-Versia module: For each feature, please select a rating from 1 to 7, where:
1 = Very Poor
2 = Poor
3 = Fair
4 = Good
5 = Very Good
6 = Excellent
7 = Outstanding

**Attribute**	**1**	**2**	**3**	**4**	**5**	**6**	**7**
Easy to navigate	○	○	○	○	○	○	○
Configurable by the user	○	○	○	○	○	○	○
Customizable	○	○	○	○	○	○	○
Well integrated / good interoperability	○	○	○	○	○	○	○
Data accuracy	○	○	○	○	○	○	○
Ability to track adherence	○	○	○	○	○	○	○
Ability to track flags and events	○	○	○	○	○	○	○
Ability to track key parameters (UF, weight, blood pressure)	○	○	○	○	○	○	○
Ability to triage patients	○	○	○	○	○	○	○
Ability to review and assess patients	○	○	○	○	○	○	○

Ability to proactively identify patient issues	○	○	○	○	○	○	○
Ability to review PD cycler data	○	○	○	○	○	○	○

Please rate the extent to which the Sharesource-Versia module useful you with the following aspects: For each statement, select a rating from 1 to 7, where:

1 = Not at all useful
2 = Slightly useful
3 = Somewhat useful
4 = Moderately useful
5 = Useful
6 = Very useful
7 = Extremely useful

**Attribute**	**1**	**2**	**3**	**4**	**5**	**6**	**7**
Supports tracking of prescriptions and program changes	○	○	○	○	○	○	○
Simplifies PD workflow	○	○	○	○	○	○	○
Saves time	○	○	○	○	○	○	○
Printing documents	○	○	○	○	○	○	○
Improves patient review and assessment (clinical & treatment data)	○	○	○	○	○	○	○
Improves documentation in Versia	○	○	○	○	○	○	○
Generates dashboards / patient summaries	○	○	○	○	○	○	○
Improves tracking of prescriptions and program changes	○	○	○	○	○	○	○
Improves tracking of adherence	○	○	○	○	○	○	○
Improves tracking of key parameters (UF, weight, blood pressure)	○	○	○	○	○	○	○
Supports discussion with patients about treatment status and progress	○	○	○	○	○	○	○

To what extent do you feel comfortable using Versia with integrated Sharesource? Please select one response:

1 = Not at all comfortable
2 = Slightly comfortable
3 = Somewhat comfortable
4 = Neither comfortable nor uncomfortable
5 = Moderately comfortable
6 = Very comfortable
7 = Extremely comfortable

What is the likelihood that you would recommend the PD Treatment module in the new version of Versia to your colleagues?
Please select one response:

1 = Extremely unlikely
2 = Very unlikely
3 = Somewhat unlikely
4 = Neither likely nor unlikely
5 = Somewhat likely
6 = Very likely
7 = Extremely likely

Thank you for sharing your experience. Your responses will contribute to the evaluation of the impact of Sharesource integration within Versia on clinical workflows and PD patient management.
